# Cell-by-Cell Dissection of Gene Expression and Chromosomal Interactions Reveals Consequences of Nuclear Reorganization

**DOI:** 10.1371/journal.pbio.0030067

**Published:** 2005-03-01

**Authors:** Brian Harmon, John Sedat

**Affiliations:** **1**University of California, San FranciscoCaliforniaUnited States of America; Stowers Institute for Medical ResearchUnited States of America

## Abstract

The functional consequences of long-range nuclear reorganization were studied in a cell-by-cell analysis of gene expression and long-range chromosomal interactions in the Drosophila eye and eye imaginal disk. Position-effect variegation was used to stochastically perturb gene expression and probe nuclear reorganization. Variegating genes on rearrangements of Chromosomes X, 2, and 3 were probed for long-range interactions with heterochromatin. Studies were conducted only in tissues known to express the variegating genes. Nuclear structure was revealed by fluorescence in situ hybridization with probes to the variegating gene and heterochromatin. Gene expression was determined alternately by immunofluorescence against specific proteins and by eye pigment autofluorescence. This allowed cell-by-cell comparisons of nuclear architecture between cells in which the variegating gene was either expressed or silenced. Very strong correlations between heterochromatic association and silencing were found. Expressing cells showed a broad distribution of distances between variegating genes and their own centromeric heterochromatin, while silenced cells showed a very tight distribution centered around very short distances, consistent with interaction between the silenced genes and heterochromatin. Spatial and temporal analysis of interactions with heterochromatin indicated that variegating genes primarily associate with heterochromatin in cells that have exited the cell cycle. Differentiation was not a requirement for association, and no differences in association were observed between cell types. Thus, long-range interactions between distal chromosome regions and their own heterochromatin have functional consequences for the organism.

## Abstract 2

The authors have devised a way to compare the expression of a gene and its association with heterochromatin in a single cell - such association tightly correlates with gene silencing

## Introduction

From the broad level of the whole chromosome down to the individual gene, interphase chromosomes in every organism studied adhere to common organizational principles (reviewed in [1]). An aspect of chromosome structure important for organism function is long-range chromosomal interactions (LRCIs) between distant loci. LRCIs have been linked with gene silencing by insulators in Drosophila [2,3] and with Polycomb silencing of homeotic genes [4,5]. LRCIs between euchromatic loci and heterochromatin can silence genes (reviewed in [6,7]). LRCIs are not static, for example, the polar organization of Drosophila embryonic chromosomes changes as homologous loci pair and LRCIs within the nucleus form [8,9,10,11]. Mouse immune cells adopt unique contacts between silenced genes and heterochromatin during differentiation and cell fate specification [12,13,14]. These changes appear to be functional rather than merely structural, such that altering LRCIs appears to have profound biological consequences.

Live studies of green-fluorescent-protein-tagged chromosomal loci reveal how LRCIs can change. Individual loci exhibit Brownian motion constrained to a defined volume, as observed in yeast [15,16,17], mammalian cells [15,18], and Drosophila [17,19]. Constraints are under developmental and cell cycle control, as evidenced by the observation that individual loci in male Drosophila pre-meiotic spermatocyte nuclei are more tightly confined in late G2 than in early G2 [19]. Relaxing constraints to allow considerable motion permits new LRCIs to form, while constraining loci more tightly can stabilize them. Developmental control of locus confinement could reconfigure a basic polar chromosomal organization into relatively stable developmental and cell-fate-specific architectures.

Drosophila position-effect variegation (PEV) is an ideal system to study the functional consequences of altered LRCIs (reviewed in [20,21]). PEV occurs when chromosome rearrangements juxtapose euchromatic genes and heterochromatin, producing a variegated expression pattern such that the gene is silenced in some but not all cells. These rearrangements also cause the affected genes to form long-range interactions with heterochromatin in a subset of cells [9,22]. Genetic evidence suggests that PEV may utilize these long-range interactions to silence genes. PEV can skip over one gene to silence another [23] or silence a wild-type locus on a homologous chromosome [9,22,24]. In the case of *bw^D^* variegation, chromosome rearrangements that alleviate PEV move the affected gene farther away from heterochromatin, while rearrangements that move the locus closer to heterochromatin enhance PEV [25,26]. This suggests that juxtaposition between a gene and heterochromatin allows for gene-to-heterochromatin interactions that can cause silencing. Once formed, these contacts may cause a gene to be silenced either by repackaging the gene into heterochromatin or by a specific silencing activity sequestered within heterochromatin itself.

Interaction with heterochromatin does seem to correlate with the silencing of specific genes, but the connection between association with heterochromatin and silencing has not been directly verified. Fluorescence in situ hybridization (FISH) techniques to identify chromosomal and heterochromatic loci are not generally compatible with the detection of gene expression. Furthermore, studies that have examined the connection between LRCIs and silencing by modulating the amount of PEV-induced repression have given conflicting results: one study found a correlation between relaxed silencing and relaxed association [22], whereas another did not [24]. Because the affected gene's expression was not compared to its association with heterochromatin on a cell-by-cell basis, it remains unclear whether, in a given cell, a heterochromatin-associated locus was silenced or expressed.

For the first time, to our knowledge, we present an experimental system that compares the expression of a variegating gene and its association with heterochromatin on a cell-by-cell basis for three different variegating genes in Drosophila whole-mount tissues. Multiple lines were chosen to ensure that results could be generally applied to PEV, rather than being limited to a specific rearrangement. The positions of variegating chromosomal loci and regions of heterochromatin were probed by FISH while fluorescent detection of eye pigments or variegating gene proteins marked gene expression. The affected gene in each line is quite far (>10 MB) from the centromere, with a block of heterochromatin placed nearby either through insertion or inversion. This provided an easy assay for long-range interactions with centromeric heterochromatin, as an interacting gene would relocate a significant distance across the nucleus to interact with centromeric regions. The variegating genes used are expressed in the Drosophila eye or eye imaginal disk; therefore, our examinations were limited to these tissues. Cell types, developmental stages, and cell cycle states were identified to determine what effects these variables might have on LRCIs with heterochromatin.

Our results show that interactions between variegating loci and heterochromatin are tightly correlated with gene silencing. Cells in which the variegating gene has been silenced exhibit a tight distribution of distances, suggesting direct interaction between heterochromatin and the distally located gene. Expressing cells, however, show a much broader distribution, with far fewer interactions between variegating loci and heterochromatin. Furthermore, these interactions with heterochromatin are primarily found in non-dividing cells in the eye disk. Differentiation is not required for LRCIs to occur, and interactions did not differ between cell types.

## Results

### LRCIs Are Strongly Correlated with Gene Silencing

**Figure 1:**
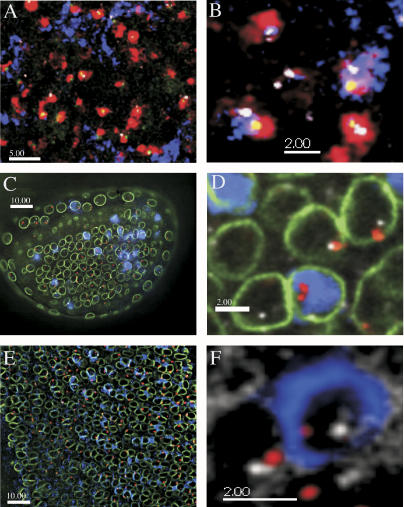
Gene Expression and LRCIs Color images show combined FISH–gene expression results for each of the three lines studied. (A and B) *bw^D^* adult eye tissue. *brown*-expressing cells with pigment are blue, DAPI is red, single-copy FISH marking the *brown* locus is white, and heterochromatic single is green. (A) Wider view of eye. (B) Close-up view showing two expressing cells at right and lower left, and one silenced cell at the lower right. (C and D) In(3L)BL1 eye disk tissue. Blue is anti-beta-galactosidase staining, green is anti-lamin staining, white is single copy FISH, red is heterochromatic FISH signal. (C) Wide-angle view of disk. (D) Close-up view showing silenced cells and expressing cells (blue in nucleus). (E and F) In(1)*rst^3^* eye disk tissue. Blue is anti-White staining, green is anti-lamin, red marks heterochromatic FISH signals, and white is single-copy FISH to the white gene. (E) Wide-angle view of disk. (F) Close-up showing expressing cell ringed by White protein and silenced cell nearby with no staining.

Using gene expression as a functional assay, the consequences of LRCIs between chromosomal loci and heterochromatin were probed on a cell-by-cell basis. The first variegating gene examined was the *brown* gene on the *bw^D^* rearrangement. Expression of *brown* RNA has only been detected in recently eclosed adult heads; therefore, gene expression experiments were confined to this tissue [27]. Our experiments were greatly simplified by working in a *scarlet* background as in [26] as this eliminated the brown pigments from the eye. Therefore *brown*-expressing cells contained red pteridine pigment while non-expressors contained none (Figure 1).

**Figure 2:**
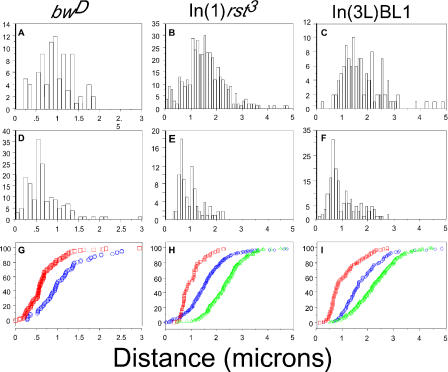
Silenced Genes Associate Tightly with Heterochromatin Physical distances in microns between heterochromatic sequences near each chromosome's centromere and the variegating gene were measured and sorted based on expressing or non-expressing cells. (A–C) Expressing cells. (D–F) Silenced cells. (G–I) percentile plots of (A–F), in which blue represents expressing cells, red represents silenced cells, and green represents wild-type nuclei. *bw^D^* nuclei are Su(*bw^D^*)208 cells in adult eyes; In(1)*rst^3^* and In(3L)BL1 data are from imaginal eye disks.

Gene-to-heterochromatin distance distributions from *brown*-expressing cells were quite different from those of silenced cells (Figure 2). The distribution of distances from expressing cells was broad, centered around 1 μm and extending to about 2 μm (Figure 2A). Looking at the cumulative percentage plots, few nuclei (10%) had variegating-gene-to-heterochromatin distances shorter than 0.5μm, and barely 25% had distances shorter than 0.75 μm (Figure 2G, blue line). Silenced cells, however, had a tight distribution of distances centered around 0.5 μm, with virtually all variegating-gene-to-heterochromatin distances being under 1 μm (Figure 2D). Cumulative percentage plots show nearly 40% of distances were shorter than 0.5 μm, and nearly 80% of nuclei had distances shorter than 0.75 μm (Figure 2G, red line). The Mann–Whitney *U* test revealed that these differences were highly significant. (*p* < 0.0001; Table 1).

**Table 1:**
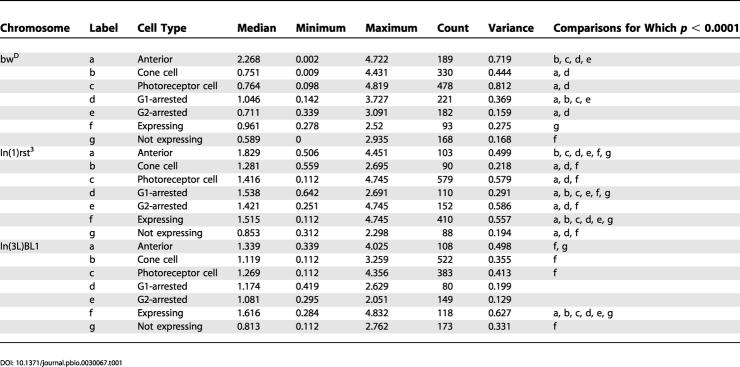
Summary of Reported Results

The second variegating gene examined was the *white* gene in the In(1)*rst^3^*. Antisera developed against the unique N-terminus of the White protein (see Figure S1 and part 1 of Protocol S1) stained *white*-expressing cells in In(1)*rst^3^* disks as expected from their eye pigmentation (Figure 1E and 1F). LRCIs between *white* and the 1.688 satellite correlated strongly with PEV-mediated silencing of *white* (Figure 2B, 2E, and 2H). As before, cells expressing the variegating gene had a broad distribution of distances, in this case centered at 1.5 μm and continuing to 4 μm (Figure 2B). Cumulative percentage plots reveal that only 20% of nuclei expressing *white* had variegating-gene-to-heterochromatin distances under 1 μm (Figure 2H, blue line). The distance distribution for silenced cells was much narrower than that for expressing cells, with distances centered around 0.8 μm and extending to only 2 μm in total distance (Figure 2E). Cumulative percentage plots show that over 60% of nuclei from silenced cells had variegating-gene-to-heterochromatin distances of under 1 μm (Figure 2H, red line) compared to 20% for expressing cells. Differences between expressing and silenced populations of cells were highly significant (*p* < 0.0001; Table 1). Despite the marked differences in association levels between expressing and silenced cells, the distributions of distances overlapped considerably between expressing and non-expressing cells (Figure 2B and 2E).

The third variegating gene examined was the heat-shock inducible beta-galacosidase transgene located on the In(3L)BL1 rearrangement. The expression of beta-galactosidase in In(3L)BL1 showed no preference for cell type, and was only found behind the morphogenic furrow (Figure 1C and 1D) [28,29]. In(3L)BL1 nuclei showed patterns of interaction and expression similar to those seen in the *brown* and *white* variegating lines. Expressing cells showed a broad distance distribution centered around 1.5 μm and extending out to nearly 4 μm (Figure 2C). Cumulative percentage plots show barely 15% of expressing cells had variegating-gene-to-heterochromatin distances 1 μm or less (Figure 2I, blue line).

In(3L)BL1 silenced nuclei displayed a rather narrow distribution, with a median distance of under 1 μm (Figure 2F). As in In(1)*rst^3^* disks, cumulative percentage plots also showed more than 60% of silenced nuclei had distances between the silenced gene and heterochromatin under 1 μm (Figure 2I, red line). The narrow distribution of distances meant that silenced nuclei rarely had variegating-gene-to-heterochromatin distances greater than 2 μm, while expressing cells had greater distances in at least 50% of cells. Also similar to In(1)*rst^3^,* variegating genes in silenced cells were not 100% associated with heterochromatin (Figure 2C and 2F), with 40% of silenced nuclei having variegating-gene-to-heterochromatin distances greater than 1 μm. Additionally, some expressing cells had variegating-gene-to-heterochromatin distances less than 1 μm, in this case roughly 10%.

### Interaction between Variegating Genes and Heterochromatin Primarily Occurs in Non-Dividing Cells

**Figure 3:**
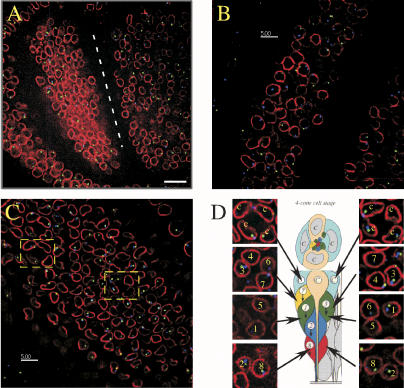
Preservation of Tissue Architecture and Cell-by-Cell Identification in the *Drosophila* Eye Disk *bw^D^* eye disks were processed for FISH and stained for nuclear lamin as described in Materials and Methods. Red, fluorescein lamin stain; blue, AACAC-cy5 heterochromatic probe; green, rhodamine-labeled P1 probe covering *brown*. (A) Low-magnification view of eye disk showing anterior (left) and posterior (right) portions of the disk. Morphogenic furrow is marked with a dotted line. (B) View of cells anterior to morphogenic furrow. These cells are still dividing and have not yet undergone differentiation. (C) Cells posterior to morphogenic furrow. These cells have ceased dividing and are differentiating into adult eye structures. (D) Four cone cell clusters from the two boxes in (C), with each photoreceptor and cone cell identified. Apical cells are at the top, basal at the bottom.

While it was clear that silencing of a variegating gene was tightly correlated with its interaction with heterochromatin, it was not known what other factors might affect this interaction. A study of how cell cycle progression and differentiation might affect interactions between a variegating gene and heterochromatin was undertaken. The cell cycle had previously been implicated as a force that periodically disrupts LRCIs [9,30]. The third-instar eye imaginal disk is an excellent tissue to explore this possibility because it contains well-separated populations of dividing and non-dividing cells. The anterior portion of the eye imaginal disk contains dividing cells (Figure 3A [left of dotted line] and 3B). The morphogenic furrow contains cells arrested in G1. Posterior to the morphogenic furrow, many cells cease dividing and differentiate (Figure 3A [right of dotted line] and 3C). Because of our sample preservation methods (see Materials and Methods), it was also possible to distinguish each individual photoreceptor cell and cone cell by their unique three-dimensional position and nuclear shape (Figure 3D; [31]). Our results therefore contain three groups of nuclei: nuclei from dividing cells anterior to the morphogenic furrow and differentiated cone cell nuclei and photoreceptor cell nuclei from behind the morphogenic furrow.

#### *bw^D^*

**Figure 4:**
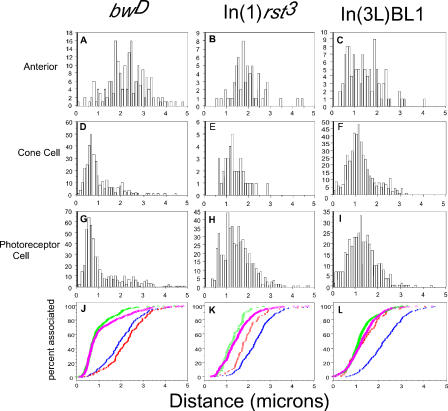
Association Primarily Occurs in Non-Dividing Cells Physical distances in microns between heterochromatic probe signals and P1 probe signals were measured for each line and sorted based upon their position relative to the morphogenic furrow and cell fate. (A–C) Cells anterior to morphogenic furrow. (D–F) Differentiated cone cells behind the morphogenic furrow. (G–I) Differentiated photoreceptor cells behind the morphogenic furrow. (J–L) Percentile plots for histograms in (A–I). Blue, wild-type cells; red, anterior cells; green, cone cells; pink, photoreceptor cells.

LRCIs were dramatically different between dividing and non-dividing cells in *bw^D^* disks (Figure 4). Cells anterior to the morphogenic furrow (Figure 4A) had a roughly Gaussian distribution of distances. The cumulative percentage plots show that few nuclei had less than 1 μm distance between the variegating gene and heterochromatin (<10%) (Figure 4J, red line).

Entirely different results were found for cone and photoreceptor cells behind the morphogenic furrow. The distributions in both cases were substantially skewed, with a long tail of distances representing cells in which *brown* was greater than 1 μm from heterochromatin (Figure 4D and 4G). Cumulative percentage plots reveal that interactions were far more common in differentiated cells behind the morphogenic furrow than in the dividing anterior cells (Figure 4J, purple and green lines). The majority of nuclei in both cone cells and photoreceptor cells had their variegating gene closer than 1 μm to the centromeric heterochromatin on 2R. Statistical tests showed that the distributions of cone cells and photoreceptor cells were significantly different from dividing anterior cells (*p* < 0.0001; Table 1) but were not significantly different from one another (*p* = 0.15). This argues that differences between cell types do not have a significant effect upon *brown*-to-centeromeric-heterochromatin LRCIs. Cone cells and photoreceptor cells are also different in shape and nuclear volume. The fact that no difference in heterochromatic association was found suggests that in these cell types nuclear shape and volume do not play a meaningful role in LRCIs with heterochromatin.

#### In(1)*rst^3^*

The inverted X chromosome In(1)*rst^3^* displayed similar results to that of *bw^D^*. Gene-to-heterochromatin distance distributions in anterior cells were nearly Gaussian (Figure 4B), such that few nuclei had variegating genes closer than 1 μm to the centromeric heterochromatin (<10%; Figure 4K, red line). Nuclei posterior to the morphogenic furrow displayed a skewed distribution similar to *bw^D^* (Figure 4E and 4H). Cone cells and photoreceptor cells displayed nearly identical distributions and were not statistically distinct (*p* = 0.0319). These distributions are markedly different from those of anterior cells (Figure 4B), with most nuclei exhibiting a shorter variegating-gene-to-heterochromatin distance (Figure 4E and 4H). The cumulative percentage plots confirm this (Figure 4K, purple and green lines), in that posterior cells routinely showed association levels 40% higher than anterior cells.

#### In(3L)BL1

The line In(3L)BL1 was a notable exception to previous results as it had an almost bimodal distribution of distances in cells anterior to the morphogenic furrow (Figure 4C). The cumulative percentage plots show dividing nuclei with variegating-gene-to-heterochromatin distances less than 1 μm (25%; Figure 4L, red line). This may be due to confounding effects of the multiply inverted balancer TM3. Gene-to-heterochromatin distance distributions from differentiated cells were not substantially skewed towards shorter distances relative to anterior cells (Figure 4F and 4I). There were few noticeable differences, as shown in cumulative percentage plots (Figure 4L, purple and green lines relative to blue). These differences were not statistically significant between cone and anterior cells (*p* = 0.0567; see Table 1) nor between photoreceptor and anterior cells (*p* = 0.6600; Table 1). Distributions from cone and photoreceptor cells were not significantly different (*p* = 0.0184; Table 1; Figure 4F and 4I), nor were there any noticeable differences between the cell types in the cumulative percentage plots (Figure 4L, purple and green lines).

Data from each variegating locus were compared with the behavior of loci on wild-type chromosomes; variegating loci behaved quite differently from those on wild-type chromosomes (see Table S1 and part 2 of Protocol S1).

### Differentiation Is Not Necessary for Association

**Figure 5:**
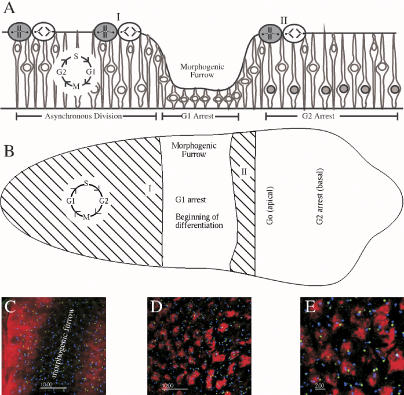
Identification of Cell Cycle Stages in *Drosophila* Eye Disks A combination of immunofluorescence to cyclins and tissue architecture was used to identify cells in specific cell cycle states. (A) Cartoon of eye disk that shows populations of dividing and cell-cycle-arrested cells. I, first mitotic wave; II, second mitotic wave. G2-arrested cells are filled nuclei located basally posterior to the furrow. (B) Cartoon of same disk viewed from above apical side of disk. Anterior is to the left. (C–E) Immunofluorescence against G2 cyclins identifies cells in G1 and G2 arrest. Red is anti–cyclin A, green is single-copy FISH signal, and blue is heterochromatic FISH signal. (C) G1-arrested cells can be identified as those near and in the morphogenic furrow that do not show G2 cyclin staining. (D) G2-arrested cells posterior to the morphogenic furrow show dense anti–cyclin A staining. (E) Close-up of cells shown in (D).

Because LRCIs were found in differentiated nuclei, this suggested that differentiation was important for interaction with heterochromatin. Alternatively, cessation of the cell cycle may allow loci to interact with heterochromatin because the periodic anaphases are eliminated [9]. The unique cell cycle profile of the eye imaginal disk presented an elegant way to distinguish between these two possibilities. Eye disk cells anterior to the morphogenic furrow divide in a band (I in Figure 5A and 5B) and arrest in the furrow at G1, as cartooned in Figure 5A and 5B. Posterior to the furrow, cells either differentiate into ommatidia or replicate their DNA. Some cells in this undifferentiated pool divide in the second mitotic wave (II in Figure 5A and 5B) and differentiate immediately. The remaining cells pause in G2 and await a signal to divide [32]. These G2-arrested cells behind the morphogenic furrow have been in interphase since the first mitotic wave ([32]; Figure 5A, shaded nuclei). If interaction requires differentiation, these G2-arrested cells should show less association than differentiated cells. If these G2 cells show equivalent levels of association, then a sufficiently long cell cycle permits variegating genes to interact with heterochromatin. G2 cells are easy to identify in third-instar eye disks as their nuclei react to anti–cyclin A antisera and are basal to differentiated and dividing nuclei (Figure 5D and 5E; [33,34]). G1-arrested cells are also clearly identified as a band of unstained nuclei between the anterior and posterior portions of the eye disk (Figure 5C).

**Figure 6:**
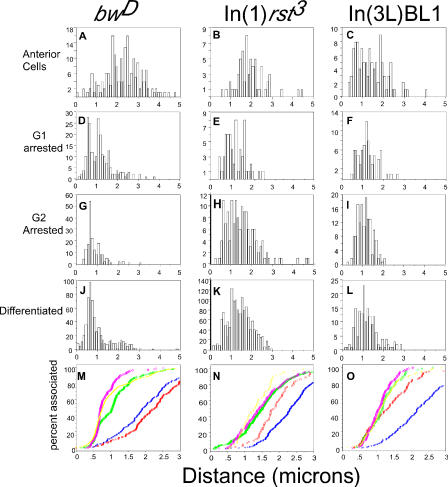
Differentiation Is Not Required for Association Physical distances in microns between heterochromatic probe signals and P1 probe signals were measured for each line and sorted based upon staining for G2 cyclins. (A–C) Cells anterior to morphogenic furrow. (D–F) G1-arrested cells (in furrow, no cyclin staining). (G–I) G2-arrested cells, basal and stained for cyclins. (J–L) Differentiated cells behind the morphogenic furrow. (M–O) Percentile plots for (A–L). Blue, wild-type cells; red, anterior cells; green, G1-arrested cells; pink, G2-arrested cells; yellow, differentiated cells.

G2-arrested cells in the *bw^D^* line showed variegating-gene-to-heterochromatin distances similar to those of differentiated cells (Figure 6). As in differentiated cells, the majority of G2-arrested nuclei had distances between the variegating gene and heterochromatin less than 1 μm. As shown in the cumulative percentage plots, G2-arrested nuclei were nearly indistinguishable from differentiated nuclei (Figure 6M, yellow and purple lines). These two distributions were also similar by statistical tests (*p* > 0.2; Table 1). Similar results were seen with In(1)*rst^3^* and In(3L)BL1 G2-arrested nuclei, such that the gene-to-heterochromatin distance distributions in arrested nuclei were indistinguishable from those of differentiated cells (Figure 4H and 4I compared to Figure 6K and 6L). Cumulative percentage plots likewise do not show any differences between arrested nuclei and differentiated ones (Figure 6N and 6O), and these distributions were not statistically distinct (*p* > 0.1; Table 1).

*bw*-to-heterochromatin distances in *bw^D^* G1-arrested nuclei displayed a bimodal distribution (Figure 6D), unlike the unimodal distributions seen for anterior cells (Figure 6A) and differentiated cells (Figure 4J). One peak shows a distance distribution similar to that seen for G2-arrested and differentiated cells (Figure 6G and 6J), while a second is intermediate between that of differentiated and anterior nuclei. Cumulative percentage plots show a marked difference between G1-arrested nuclei and all other populations, including an inflection in the curve between the two peaks in the G1 distribution (Figure 6M, red line). It appears that the morphogenic furrow is a transition state, whereby variegating genes that were once quite far from heterochromatin (Figure 6A) begin to associate with centromeric regions on the same chromosome. Similar behavior is also seen in In(1)*rst^3^* and In(3L)BL1 nuclei (Figure 6E and 6F). Distributions in these two lines are not bimodal, but they are intermediate between anterior and differentiated nuclear distributions. Cumulative percentage plots also show distinctions between these nuclei and the other populations (Figure 6N and 6O, red lines). Statistical tests showed that all three distributions of G1 nuclei were distinct from anterior cells (*p* < 0.0001; Table 1), but that only *bw^D^* G1 nuclei were different from differentiated cells (*p* < 0.0001; Table 1).

### Variegating Genes Associate with Heterochromatin at Their Own and Other Centromeres

**Figure 7:**
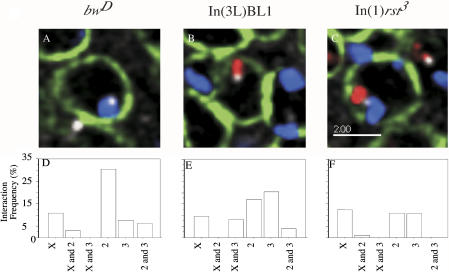
Interaction of Variegating Genes with Heterochromatin on Other Chromosomes All three lines were examined to see how often variegating genes interacted with heterochromatin on their own and other chromosomes. (A) Close-up of In(1)*rst^3^* nucleus showing variegating gene interaction with heterochromatin on its own chromosome. (B) Close-up of In(1)*rst^3^* nucleus showing variegating gene interaction with heterochromatin on a different chromosome. (C) In(1)*rst^3^* nucleus showing variegating gene interaction with heterochromatin on both its own and another chromosome. (D–F) Bar plots of interaction frequencies of variegating genes with their own heterochromatin and that found on other chromosomes. A distance of 1.0 μm was chosen as the maximum distance for interaction to occur.

An appreciable percentage of loci were found far from centromeric *cis-*heterochromatin, even in PEV-silenced nuclei. These silenced yet far loci may form LRCIs with heterochromatin on other chromosomes (*trans*-heterochromatin). While other authors found that variegating rearrangements involving *bw* preferentially associated with *cis-*heterochromatin [9,35], we felt this issue merited reexamination. In two sets of experiments per line, probes marking variegating loci were compared with two different heterochromatic probes: one marked *cis-*heterochromatin while another identified *trans*-heterochromatin. Each variegating gene was examined for promiscuous interaction with centromeric heterochromatin on each large chromosome. Data were sorted based on whether the variegating gene was within 1 μm of heterochromatin on its own chromosome (Figure 7A), on a different chromosome (Figure 7B), or on both chromosomes (Figure 7C).

As in Dernburg et al. [9], the *brown* locus on the *bw^D^* chromosome interacted primarily with heterochromatin on the same chromosome (Figure 7D). Interactions with *trans*-heterochromatin on Chromosomes X and 3 where the *brown* gene was not associating with *cis*-heterochromatin were generally limited to 5%–10% of examined nuclei. Interactions where the *brown* gene was close to heterochromatin on multiple chromosomes were also infrequent, between 2%–5% of examined nuclei.

The In(3L)BL1 chromosome differed from the *bw^D^* chromosome in that comparable levels of association were observed between *cis-* and *trans*-heterochromatin (Figure 7E). The In(3L)BL1 chromosome's transgene interacted similarly with heterochromatin on Chromosomes 2 and 3 (20% of nuclei examined), but only 10% with the X chromosome. Interactions of the variegating gene with heterochromatin on multiple chromosomes were less common, at around 5%–10% of nuclei examined. The variegating locus on In(3L)BL1 seemed to show a preference for associating either with heterochromatin on its own chromosome or that of Chromosome 2.

The In(1)*rst^3^* line showed similar levels of association with heterochromatic blocks on all three chromosomes, with around 10% of nuclei exhibiting distances less than 1 μm (Figure 7F). Unlike in the other lines examined, the variegating gene was rarely close to heterochromatin on multiple chromosomes, suggesting that the *white* locus interacts with only one block of heterochromatin at a time.

While the *bw^D^* chromosome exhibited a marked preference for associating with heterochromatin on its own chromosome, the other two lines did not. This suggests that the tails of the distances seen in the silenced cells of In(1)*rst^3^* and In(3L)BL1 could well be explained by interaction with heterochromatin on other chromosomes. *bw^D^'*s infrequent association with blocks of heterochromatin on other chromosomes may explain the smaller tails seen in the distributions of *bw^D^* disk nuclei.

## Discussion

The variegating rearrangements studied here cause a nuclear reorganization with clear consequences for the organism. The rearrangements alter the behavior of affected loci relative to wild-type (see Table S1 and part 2 of Protocol S1), changing their nuclear position. This in turn increases the likelihood that nearby genes will contact and form persistent interactions with heterochromatin. Those loci that do interact with heterochromatin may be silenced. Supporting this conclusion is the observation that variegating-gene-to-heterochromatin distance distributions in silenced cells are vastly different from those of expressing cells (Figure 2D–2F). Silenced nuclei show a tight unimodal distribution with a peak centered around short distances, 0.5 μm in the case of *bw^D^*, and 0.8 μm in the case of In(3L)BL1 and In(1)*rst^3^*. We posit that the lower variability of these silenced cell distributions means that variegating genes are forming persistent contacts with heterochromatin. Because the same level of association was seen between recently differentiated nuclei and those that had differentiated as many as 12–18 h before (see Figure S2 and part 5 of Protocol S1), this suggests that the interactions are quite persistent and do not appear and disappear periodically. Distances of nearly a micron may seem inordinately high. However, since the analysis scheme measured between FISH signal centers, distances will never be zero even when FISH signals overlap considerably. This is compounded by the relatively large volume occupied by heterochromatic FISH signals. However, FISH signals that we classified as interacting based on distance were often touching or partially overlapping (Figure 1). Unlike silenced cells, expressing cells have a broader, nearly Gaussian distribution centered at 1.0–1.5 μm and extending to nearly 4 μm (Figure 2A–2C). We interpret the more variable distribution to mean that expressing loci are not interacting with heterochromatin and are therefore less restricted.

Despite clear differences between expressing and silenced loci behavior, we do not see absolute distinctions between them. Appreciable numbers of silenced loci are far from *cis-*heterochromatin, while some expressing loci are quite close. Some of these results may be explained by limitations of our experimental procedures. Not all centromeric heterochromatin is labeled by our FISH methods. It remains possible that a “far silenced” gene may be interacting with heterochromatin not labeled by our oligonucleotide probes in either *cis-* or *trans*-heterochromatin. Two lines exhibited significant association with *trans*-heterochromatin, suggesting that this could explain cases where silenced genes are not associated with labeled *cis-*heterochromatin. Some of the expressing yet heterochromatin-close cases may have expressed the gene for some time before contact with heterochromatin silenced the variegating locus. Our use of accumulated gene product to mark gene expression would mask these examples. While FISH methods do not drastically perturb nuclear structure, these methods may impact interloci distances somewhat [36,37]. Heat shock utilized for the In(3L)BL1 line may explain its unique behavior relative to other chromosomes, as heat shock has been shown to affect nuclear structure [2]. A live chromosomal imaging system [15] that tracks the long-range interactions of a variegating gene with heterochromatin should provide the best control for sample manipulations discussed here.

Specific biological interactions may also explain the “far silenced” and “close expressing” loci. Interaction with heterochromatin may be required for the establishment but not the maintenance of silencing. Once a gene is silenced, chromosomal motion may pull it away from centromeric heterochromatin without changing the affected gene's expression. A second possibility is that the proximal block of heterochromatin near the variegating locus can occasionally silence the gene by itself. For example, the block of heterochromatin near the *white* gene in In(1)*rst^3^* is known to be a weak silencer [38]. The observation that variegating loci in some expressing cells seem to interact with heterochromatin (upper limit of 20%) may mean that interaction does not guarantee silencing. Early transcriptional activation allows a gene to escape PEV, arguing that once a gene is expressed, future interactions with heterochromatin might not silence it [39]. The timing of interaction relative to the normal expression pattern of the gene may therefore be critical. Some genes vary in their sensitivity, such that interaction with heterochromatin may not necessarily result in silencing [40,41].

Based on Figure 2 and the results in Table 1, we can determine what the differences between expressing and silenced cells mean in terms of chromosomal and nuclear dimensions. In silenced cells, the median distance between the variegating locus and heterochromatin is nearly halved relative to expressing cells. For example in In(1)*rst^3^* the distance falls from around 1.5 μm in expressing cells to 0.85 μm in silenced ones. Based upon calculations of linear base pair distance, this is what we would expect if the silenced locus is located 9 MB closer to heterochromatin than in the expressing case, or about half of a chromosome arm. Given the complex folding of an interphase chromosome arm, this reduction is likely more pronounced than a linear calculation would suggest. Additionally, the behavior of a silenced locus is different in ways far more dramatic than merely “closeness” to heterochromatin. The distribution of distances for silenced loci are also less variable than for expressing loci, roughly half that of an expressing locus. This becomes particularly clear when we imagine the variegating-gene-to-heterochromatin distance in statistical terms, using two standard deviations as a 95% confidence interval. Using the In(1)*rst^3^* case as an example, the expressing locus might be anywhere from 0 to 3 μm from heterochromatin, while the silenced locus will not be found farther than 1.7 μm away. Thinking about this in terms of three-dimensional nuclear volume, this means than an expressing locus will inhabit a 95% confidence volume five times larger than that of a silenced locus. We posit that the shorter median distance and reduced variability in position relative to heterochromatin suggests a persistent interaction.

It is not known to what extent the association of a variegating gene with heterochromatin is a cause or an effect of gene silencing. Even though our results argue that the two events are connected, a gene could be silenced by local factors and associate with heterochromatin independently. Future examinations of this problem would require a live study to pinpoint precisely when interactions occur. If interaction occurs before the gene is silenced, that suggests that the interaction could be a cause of silencing. If interactions occur after silencing, however, this would suggest that the interaction is merely a side effect. Studying the persistence of such associations will reveal whether interaction with heterochromatin causes loci to be more constrained than non-interacting loci, which could explain why gene-to-heterochromatin distance distributions from expressing cells are so much broader than those of silenced cells.

Now that specific interactions with heterochromatin have been shown to be closely correlated with silencing, one can ask how such interactions form. Time-lapse studies have shown that individual loci can move substantial distances within the nucleus, eventually bringing the variegating locus into contact with heterochromatin [19]. Additional live studies found that chromosomal loci can be held in place after contacting certain structures such as the nuclear envelope or the nucleolus [42]. Once contact is made, the proximal and centromeric blocks of heterochromatin may mediate a persistent interaction through multimerization of proteins such as HP1. Loci on wild-type chromosomes will not form persistent interactions because they lack a proximal block of heterochromatin. Interestingly, studies have found that as interphase progresses, nuclear motion decreases such that contacts between euchromatic loci and heterochromatin may stabilize [19,43,44].

Recent insights into the molecular behavior of heterochromatin proteins suggest how persistent interaction with heterochromatin may silence genes (reviewed in [45]). We hypothesize that the associated loci, because of intimate (possibly molecular) contact, acquire the molecular features of heterochromatin. The chromatin of PEV-silenced genes acquire heterochromatic features such as a regular nucleosome array, insensitivity to nucleases [46], and binding of heterochromatin proteins [47,48]. This altered chromatin structure seems to occlude the affected gene's promoter, preventing the loading of RNA polymerase and transcriptional activators, thereby preventing gene expression [49].

**Figure 8:**
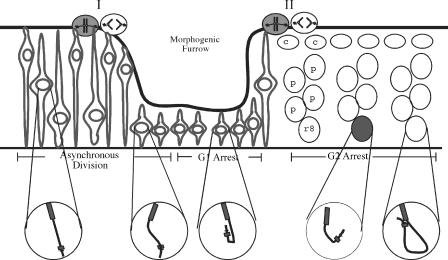
Model of Variegating Gene Association with Heterochromatin and Its Consequences Cartoon of eye disk showing when and where association occurs and its effects on gene silencing. Anterior is to the left. Long-range interactions are not observed anterior to the morphogenic furrow in regularly dividing cells. In the morphogenic furrow, G1 arrest allows variegating genes the time to begin to find and associate with blocks of heterochromatin. Once behind the morphogenic furrow, variegating loci in more cells associate with heterochromatin, and once formed these interactions persist. When a developmental signal is sent for a gene to be expressed, its association with heterochromatin greatly affects its chances for being expressed, such that a gene interacting with heterochromatin is far less likely to be activated than one that is not.

A model of how LRCIs with heterochromatin occur in the eye disk is outlined in Figure 8. In dividing cells anterior to the morphogenic furrow, few loci interact with heterochromatin. As cells pass into the morphogenic furrow, the G1 cell cycle arrest allows loci to explore their chromosome territory and make contacts with regions of heterochromatin on their own and possibly other chromosomes. Loci that contact heterochromatin may form persistent interactions that cause silencing. The normal expression pattern of the *white* gene, for example, is now altered by association with heterochromatin. Instead of being expressed in every r8 cell behind the morphogenic furrow, as in a wild-type chromosome, its expression pattern is periodically interrupted by silenced cells. Loci that contact but do not form persistent interactions remain relatively unconstrained.

This is the first study to examine the expression state of a chromosomal locus and its interaction with heterochromatin on a cell-by-cell basis within intact tissue. In each of the three cases examined, relocalization of a gene to heterochromatin was tightly correlated with gene silencing. These results strongly suggest that spatial organization of the Drosophila genome is an integral part of organism function.

## Materials and Methods

### 

#### Drosophila strains and chromosomes

Three classes of rearrangements were used, each representing one of *Drosophila's* three largest chromosomes: X, 2, and 3. Each variegates for a different gene (Table 2) and has a proximal block of heterochromatin placed into or nearby the variegating locus. It was expected that the small block of heterochromatin near the variegating gene would mediate interaction between the variegating gene and centromeric heterochromatin without silencing the variegating gene directly.

**Table 2:**
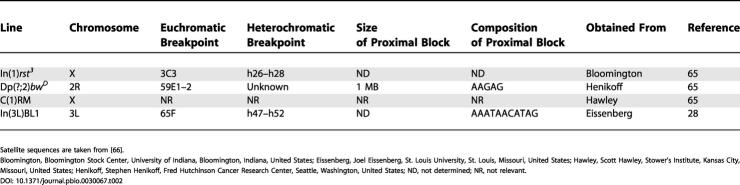
List of Rearranged Chromosomes Studied

Satellite sequences are taken from [66]

Bloomington, Bloomington Stock Center, University of Indiana, Bloomington, Indiana, United States; Eissenberg, Joel Eissenberg, St. Louis University, St. Louis, Missouri, United States; Hawley, Scott Hawley, Stower's Institute, Kansas City, Missouri, United States; Henikoff, Stephen Henikoff, Fred Hutchinson Cancer Research Center, Seattle, Washington, United States; ND, not determined; NR, not relevant

The X chromosome was represented by the line In(1)*rst^3^ y^1^car^1^*. Variegation for *white* was produced by mating In(1)*rst^3^* males with virgin females of the genotype C(1)RM *v, v;* C(4) *ci ev^R^* × X^Y, In(1)EN, *v f B;* C(4) *ci ev^R^* to produce normal phenotype XX/Y females and variegating X/O males [50]. X/O males were distinguished from XX/Y females by the expression pattern of *white* in third-instar eye disks (see Results).

The second chromosome was represented by two different lines containing the *bw^D^* insertion [51]. Experiments studying the *bw^D^* rearrangement in imaginal disk tissue were performed with larvae homozygous for the *bw^D^* chromosome. Control experiments with *bw^D^*/*bw^+^* tissues produced identical results (data not shown). Experiments studying the long-range interactions with heterochromatin and silencing in adult eyes used the line Su(*bw^D^*)208/SM1. The In(2LR)SM1, *Cy* balancer carries a wild-type *brown* gene to allow *bw* variegation to be observed. The Su(*bw^D^*)208 rearrangement increased the number of expressing cells per eye from the normal *bw^D^*/*bw^+^* expression level (<5%) to about 20%–30% [26]. Experiments were carried out in a *scarlet (st)* background to eliminate brown ommochrome pigment; *brown*-expressing cells appeared red against a field of white non-expressors, as opposed to red-brown expressors against a field of brown non-expressors. All gene expression experiments in adult eyes were in flies with the genotype In(2LR)SM1 *Cy*/Su(*bw^D^*)208; *st*/*st*.

The third chromosome was represented by the beta-galactosidase variegating line In(3L)BL1 [28]. The precise genotype for the In(3L)BL1 line is Df(1)*y w,* In(3L)BL1/Tm3 *hb-lacZ*.

**Figure 9:**
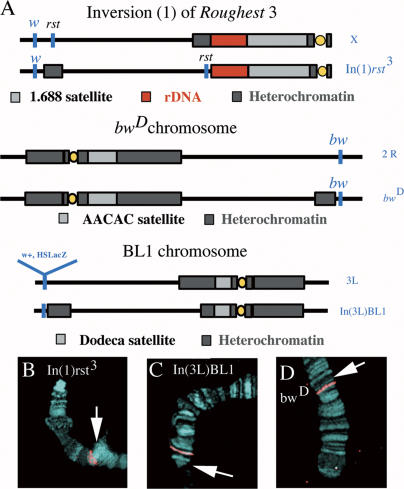
Variegating Lines and Probes Used in This Study Variegating lines were used for three different chromosomes, each variegating for a different gene. For full details see Materials and Methods. (A) Diagram of each line showing approximate breakpoints and locations of variegating genes. FISH probes were made from P1 clones covering each gene and from heterochromatic sequences unique to each chromosome (see Materials and Methods). (B–D) FISH probes for each chromosome. FISH probes were hybridized to polytene squashes to show cytological location of each probe. Regions of proximal heterochromatin from inversion and insertion are marked with an arrow. Each chromosome spread is taken from individual experiments. (B) Probe used in experiments for the line In(1)*rst^3^*. (C) Probe used in experiments for the line In(3L)BL1. (D) Probe used in experiments for the line *bw^D^*.

Diagrams of all variegating chromosomes including FISH staining of polytene chromosomes are shown in Figure 9.

#### FISH probes

FISH probes marked the three-dimensional locations of chromosomal loci. Repetitive heterochromatin sequences were hybridized with hapten-conjugated DNA oligonucleotide probes. Each probe's location is presented in Figure 9A. Chromosome 3 heterochromatin was hybridized to a 46-bp DNA oligonucleotide containing the sequence ( CCCGTACTGGT)_4_ corresponding to the dodeca satellite sequence. Chromosome 2 heterochromatin was hybridized to a 35-bp DNA oligonucleotide containing the sequence ( AACAC)_7_. X heterochromatin was hybridized to a PCR product of 359 bp comprising the bulk of the 1.688 satellite sequence. Satellite sequences and probe construction are discussed in more detail in [52]. DNA oligonucleotides were synthesized by Invitrogen (Carlsbad, California, United States) using sequences as published in [52]. Heterochromatic probes made from these oligonucleotides were labeled with digoxygenin-conjugated nucleotides by terminal transferase. Digoxygenin-labeled probes were detected by sheep anti-dig F(ab)2 fragments coupled to Cy5 (Amersham Biosciences, Piscataway, New Jersey, United States).

Euchromatic probes were made from P1 phage clones provided by the Berkeley Drosophila Genome Project and Genome Systems (St. Louis, Missouri, United States) [53,54]. Experiments with *bw^D^* lines used P1 clone DS003480, which hybridizes to 59E (Figure 9B) [9]. Experiments with the In(1)*rst^3^* line used P1 clone DS006812, corresponding to 3C2–3. For the line In(3L)BL1, the P1 clone DS000374 was utilized, which hybridizes to 65D2–3. P1 DNA was amplified in bacteria and purified via alkaline lysis and silica gel chromatography (Qiagen Midi Tips, Qiagen, Valencia, California, United States). The purified DNA was digested with restriction endonucleases and labeled with Rhodamine 4-dUTP (FluoroRed, Amersham Biosciences) by terminal transferase as described previously [52].

#### Sample preparation

Eye imaginal disks were dissected from climbing third-instar larvae raised at 16 °C and fed on a yeast paste/glucose/agar/instant Drosophila food (Carolina Biological Supply, Burlington, North Carolina, United States) recipe. Eye disks were dissected into modified M3 medium [55] or Grace's and fixed for 15 min in 3.7% formaldehyde and Buffer A+ [56]. After fixation, disks were permeablized for 30 min in Buffer A+/0.1% Triton X-100 (Pierce Chemical, Rockford, Illinois, United States) and transferred to PBS (pH 7.4) with 0.1% Tween 20, unless specified otherwise.

Adult eyes from recently eclosed (0–3 h) adult heads were dissected into a drop of Grace's medium and fixed for 15 min in Buffer A+/3.7% formaldehyde. Heads were transferred back into Grace's medium and the eyes cut away with a 15° microsurgical knife (Surgical Specialties Corporation, Reading, Pennsylvania, United States). These eyes were fixed for an additional 15 min in A+/3.7% formaldehyde and then pried away from the compound lens with forceps and a scalpel modified into a scoop (made by Daron Brown, Fine Science Tools, Foster City, California, United States). Bovine serum albumin (5%) prevented eyes from sticking together.

#### Detection of gene expression

Detection of gene expression in In(3L)BL1 relied on immunofluorescence of the beta-galactosidase gene product after heat shock at 37 °C. Eppendorf tubes containing a 1-ml agarose plug were preheated for one hour at 37 °C in a circulating water bath. The plug was removed, larvae were placed inside the eppendorf tube, and the pre-warmed plug was loosely placed over the larvae and the tube sealed. Larvae were incubated in the water bath for 5 min, followed by immersion in a room temperature water bath and a 1 h recovery period. Eye disks were dissected, followed by blocking for 4 h in PBS +0.1% Tween 20 (PBT) plus 5% dry milk (Nestle, Solon, Ohio, United States). Disks were incubated with a mouse monoclonal antibody in PBT at a dilution of 1:1,000 (Promega, Madison, Wisconsin, United States) for 2 h. After three 30-min washes in PBT, disks were incubated with horseradish-peroxidase-conjugated anti-mouse secondary antibodies (Jackson ImmunoResearch, West Grove, Pennsylvania, United States) at a 1:500 dilution in PBT for 1 h. After three more washes in PBT, the horseradish-peroxidase-coupled antibody was treated with fluorescent coumarin-coupled tyramide signal amplification reagents (TSA-direct, PerkinElmer Life Sciences, Wellesly, Massachusetts, United States) to develop a stable fluorescent stain that would survive FISH.

Detection of gene expression in the line In(1)*rst^3^* relied on immunofluorescence to the *white* gene product. Details of anti-White antibodies are presented in Figure S1 and part 1 of Protocol S1. Anti-White antibody staining proceeded as anti-beta-galactosidase staining, except that anti-White primary antibody was used at a concentration of 0.5 μg/ml in PBT and stained overnight. The primary antibody was detected by anti-rabbit horseradish-peroxidase-conjugated secondary antibodies (Jackson ImmunoResearch) at dilution of 1:500, followed by treatment with tyramide as described above.

For *bw^D^,* adult eyes were embedded in polyacrylamide immediately after dissection (see below), stained with 0.5 μg/ml DAPI in PBS (pH 7.4), and imaged at low magnification with an Olympus (Tokyo, Japan) 20 × 0.8 NA lens to identify gross structural features for later realignment. Select regions of each eye were imaged under a 1.4 NA 100x lens to acquire high-resolution three-dimensional images of each nucleus. The precise positions of both high and low magnification datasets were recorded for realignment after FISH. Expressing cells were identified by their bright autofluoresence.

#### Detection of cell cycle arrest

Experiments to determine the role of differentiation and the cell cycle were carried out in third-instar eye disks. A rabbit anti–cyclin A antibody detected the presence of G2 cyclins [34]. G1-arrested cells were identified by their location within the morphogenic furrow and the absence of cyclin A.

#### FISH

Whole-mount disks were processed for in situ hybridization as described elsewhere [52]. After FISH, tissues were stained in 2XSSCT with anti-Lamin antibodies [57] and an FITC-labeled goat anti-mouse secondary antibody to mark the nuclear periphery (Jackson ImmunoResearch). Before imaging, all tissues were washed three times in 50 mM Tris (pH 8.0) and embedded in polyacrylamide pads as in [58] to support three-dimensional structure.

Adult eyes were processed for FISH similar to [58] with modifications described below. Adult eyes were placed within a nail polish ring on a #1.5 20 × 30 mm coverslip in 13.5 ml of Tris (pH 8.0). Then 6.5 ml of 3X activated acrylamide buffer (Buffer A+, 15% acrylamide, and 0.333% bis-acrylamide) was added. Twenty-five microliters of 20% ammonium persulfate and 25 μl of 20% sodium sulfite were added to 500 μl of acrylamide buffer immediately before addition to tissue. The acrylamide buffer drop was mixed by repeated pipetting, and sealed with a clean 20 × 30 mm coverslip. After 30 min of polymerization, the top coverslip was removed and pads washed four times in Buffer A+, followed by DAPI staining and pigment imaging.

After pigment imaging, pads were stepped into 2XSSCT/50% formamide and incubated with probe and hybridization solution overnight at 37 °C before denaturation. Pre- and post-FISH washes proceeded as described for eye disks in PCR tubes, except that washes were extended to 1 h apiece in humid chambers with agitation. Eyes were denatured as described elsewhere [52,58], except that denaturation was extended to 10 min. After FISH, eyes were washed five times with 50% formamide/2xSSCT, stepped into PBT, and stained with DAPI.

#### Three-dimensional imaging

Before mounting, all tissues were washed three times in 50 mM Tris (pH 8.0) to remove any detergent. Disks were mounted in Vectashield (Vector Labs, Burlingame, California, United States) while adult eyes were mounted with ProLong (Molecular Probes, Eugene, Oregon, United States). Three-dimensional imaging was carried out on a computer-controlled epifluorescence microscope [59,60]. All images were collected on a Peltier-cooled CCD camera (Photometrics, Tucson, Arizona) connected to an SGI Indigo2 workstation (SGI, Mountain View, California, United States). Imaginal disks were imaged with a 60x Olympus 1.4 NA oil lens while adult eyes were imaged on an Olympus 20 × 0.8 NA oil lens followed by a Nikon (Tokyo, Japan) 100 × 1.4 NA oil lens. For adult eyes, the same tissues examined for pigmentation were re-imaged and carefully aligned with pre-FISH images. Data were processed after collection by a constrained iterative deconvolution software program [61].

#### Image analysis

Image analysis was performed with the Image Visualization Environment software package PRIISM ([62]; D. Diggs and E. Branlund, unpublished data). The Water algorithm automatically identified nuclei [63] and segmented them into three-dimensional objects. After segmentation the center point of each object was the starting point to find the nuclear periphery. The nuclear lamin signal was found by searching for pixels above an intensity threshold a specified radial distance from the center point. Pixels above the threshold were marked and refined by a second- and third-order surface harmonic expansion [10]. Individual FISH signals within each modeled nucleus were identified based on their intensity peak above background (M. Lowenstein and D. Diggs, unpublished data). FISH signal distances were measured and sorted by nucleus membership. Distances were measured from the intensity-weighted center of mass rather than from the edge of each FISH signal. This means that even overlapping FISH signals will return a measured distance. Differences between different groups of nuclear distances were tested for statistical significance by the Mann–Whitney *U* test (see Table 1), which is a two-sample rank-sum test for position that makes no assumptions regarding the distributions of sample data [64].

Plots and statistical comparisons were performed by the Statview 4.5 (Abacus Concepts, Berkeley, California, United States) analysis program. Results are summarized in Table 2.

#### Data presentation

Groups of distances mentioned previously were tested for statistical significance relative to one another (see Table 1). Variegating chromosomal loci close to heterochromatin were considered to be interacting, while loci farther away were considered to be not interacting. Data were presented in two different ways. First, standard histograms presented the full distribution of the data including any subpopulations. Second, cumulative percentile plots with percent association between variegating genes and their own centromeric heterochromatin plotted as a function of gene-to-heterochromatin distance more clearly distinguished varying interaction levels between different cell populations. This plotting method also revealed that the exact placement of a cutoff for interaction between loci was unimportant, as clear differences could be seen at distances less, equal to, or greater than 1 μm. As a general rule 1 μm in distance was used as a cutoff for interaction. This cutoff was not intended to be mechanistically meaningful, but to serve as a rule of thumb for thinking about the results.

#### Monte Carlo analysis and correlation between nuclear distances and nuclear dimensions

Distance distributions in eye disk nuclei were compared to pairwise distances measured from 50 points randomly placed within the nucleus. Also, distances between chromosomal loci were examined for their sensitivity to nuclear shape and volume. Data and results are presented in Table S2 and parts 3 and 4 of Protocol S1.

## Supporting Information

Protocol S1 Anti-White Antibody Generation, and the Effects of Gene Expression, Nuclear Dimensions, and Time on Gene to Heterochromatin Distances (31 KB DOC).

Figure S1 Production and Characterization of Anti-White Antisera Full details available in Materials and Methods.
(A) Western blots showing specificity of antibody for a single polypeptide of approximately 70 kDa in larval (L) and adult (A) extracts. Preimmune sera show no reactivity for that polypeptide.
(B) Immunofluorescence of eye disks with affinity-purified anti-White antisera. Anti-White staining surrounded each nucleus, possibly because the protein is enriched in the endoplasmic reticulum. Expression was seen only in four cone cell ommatidial clusters several cell rows behind the morphogenic furrow.
(C) Immunofluoresence in eye disks of the *white* variegating line In(1)*w^m51b^*. Gaps in the *white* expression pattern are marked with dotted polygons.
(D) Vertical series of images through a single four cone cell cluster in the eye disk stained for White protein (cyan) and the nuclear lamin (green). In every cell cluster examined, expression was only seen in the eighth photoreceptor. Each panel represents a step of about 2.5 μm from the most apical to basal parts of the cluster. Total distance from basal edge of cell cluster to cone cells is about 10 μm.
(2.9 MB TIF).

Figure S2 Comparison of Different Cell Populations Based upon Time after Differentiation or Cell Cycle Arrest Individual rows of differentiated nuclei were compared to one another to see whether differences in interaction levels existed between younger and older cells.
(A–C) Row-by-row analysis method for lines *bw^D^*, In(1)*rst^3^,* and In(3L)BL1 respectively. Each color represents a different row, rows 4–8 respectively.
(D–F) Scattergrams for each row based on pooling of data from three different disks per line. Y-axis is distance in microns; X-axis is row number.
(G–I) Percentile plots of each row.
(2.6 MB TIF).

Table S1 Variegating Locus-to-Heterochromatin Distances in Wild-Type Cells and Random Distributions (53 KB DOC).

Table S2 Effects of Nuclear Dimensions on Variegating Gene-to-Heterochromatin Distances (40 KB DOC).

## Abbreviations

FISH: fluorescence in situ hybridization
LRCI: long-range chromsomal interaction
PEV: position-effect variegation

## References

[plbi-03-03-12-b01] Marshall WF (2002). Order and disorder in the nucleus. Curr Biol.

[plbi-03-03-12-b02] Gerasimova TI, Byrd K, Corces VG (2000). A chromatin insulator determines the nuclear localization of DNA. Mol Cell.

[plbi-03-03-12-b03] Muller M, Hagstrom K, Gyurkovics H, Pirrotta V, Schedl P (1999). The mcp element from the Drosophila melanogaster bithorax complex mediates long-distance regulatory interactions. Genetics.

[plbi-03-03-12-b04] Messmer S, Franke A, Paro R (1992). Analysis of the functional role of the Polycomb chromodomain in Drosophila melanogaster. Genes Dev.

[plbi-03-03-12-b05] Franke A, Messmer S, Paro R (1995). Mapping functional domains of the Polycomb protein of Drosophila melanogaster. Chromosome Res.

[plbi-03-03-12-b06] Gasser SM (2001). Positions of potential: Nuclear organization and gene expression. Cell.

[plbi-03-03-12-b07] Cockell M, Gasser SM (1999). Nuclear compartments and gene regulation. Curr Opin Genet Dev.

[plbi-03-03-12-b08] Hiraoka Y, Dernburg AF, Parmelee SJ, Rykowski MC, Agard DA (1993). The onset of homologous chromosome pairing during Drosophila melanogaster embryogenesis. J Cell Biol.

[plbi-03-03-12-b09] Dernburg AF, Broman KW, Fung JC, Marshall WF, Philips J (1996). Perturbation of nuclear architecture by long-distance chromosome interactions. Cell.

[plbi-03-03-12-b10] Marshall WF, Dernburg AF, Harmon B, Agard DA, Sedat JW (1996). Specific interactions of chromatin with the nuclear envelope: Positional determination within the nucleus in Drosophila melanogaster. Mol Biol Cell.

[plbi-03-03-12-b11] Fung JC, Marshall WF, Dernburg A, Agard DA, Sedat JW (1998). Homologous chromosome pairing in Drosophila melanogaster proceeds through multiple independent initiations. J Cell Biol.

[plbi-03-03-12-b12] Brown KE, Baxter J, Graf D, Merkenschlager M, Fisher AG (1999). Dynamic repositioning of genes in the nucleus of lymphocytes preparing for cell division. Mol Cell.

[plbi-03-03-12-b13] Brown KE, Guest SS, Smale ST, Hahm K, Merkenschlager M (1997). Association of transcriptionally silent genes with Ikaros complexes at centromeric heterochromatin. Cell.

[plbi-03-03-12-b14] Grogan JL, Mohrs M, Harmon B, Lacy DA, Sedat JW (2001). Early transcription and silencing of cytokine genes underlie polarization of T helper cell subsets. Immunity.

[plbi-03-03-12-b15] Robinett CC, Straight A, Li G, Willhelm C, Sudlow G (1996). In vivo localization of DNA sequences and visualization of large-scale chromatin organization using lac operator/repressor recognition. J Cell Biol.

[plbi-03-03-12-b16] Straight AF, Marshall WF, Sedat JW, Murray AW (1997). Mitosis in living budding yeast: Anaphase A but no metaphase plate. Science.

[plbi-03-03-12-b17] Marshall WF, Straight A, Marko JF, Swedlow J, Dernburg A (1997). Interphase chromosomes undergo constrained diffusional motion in living cells. Curr Biol.

[plbi-03-03-12-b18] Li G, Sudlow G, Belmont AS (1998). Interphase cell cycle dynamics of a late-replicating, heterochromatic homogeneously staining region: Precise choreography of condensation/decondensation and nuclear positioning. J Cell Biol.

[plbi-03-03-12-b19] Vazquez J, Belmont AS, Sedat JW (2001). Multiple regimes of constrained chromosome motion are regulated in the interphase Drosophila nucleus. Curr Biol.

[plbi-03-03-12-b20] Elgin SC (1996). Heterochromatin and gene regulation in Drosophila. Curr Opin Genet Dev.

[plbi-03-03-12-b21] Wakimoto BT (1998). Beyond the nucleosome: Epigenetic aspects of position-effect variegation in Drosophila. Cell.

[plbi-03-03-12-b22] Csink AK, Henikoff S (1996). Genetic modification of heterochromatic association and nuclear organization in Drosophila. Nature.

[plbi-03-03-12-b23] Talbert PB, Henikoff S (2000). A reexamination of spreading of position-effect variegation in the white-roughest region of Drosophila melanogaster. Genetics.

[plbi-03-03-12-b24] Sass GL, Henikoff S (1999). Pairing-dependent mislocalization of a Drosophila brown gene reporter to a heterochromatic environment. Genetics.

[plbi-03-03-12-b25] Henikoff S, Jackson JM, Talbert PB (1995). Distance and pairing effects on the brownDominant heterochromatic element in Drosophila. Genetics.

[plbi-03-03-12-b26] Talbert PB, LeCiel CD, Henikoff S (1994). Modification of the Drosophila heterochromatic mutation brownDominant by linkage alterations. Genetics.

[plbi-03-03-12-b27] Dreesen TD, Johnson DH, Henikoff S (1988). The brown protein of Drosophila melanogaster is similar to the white protein and to components of active transport complexes. Mol Cell Biol.

[plbi-03-03-12-b28] Lu BY, Bishop CP, Eissenberg JC (1996). Developmental timing and tissue specificity of heterochromatin-mediated silencing. EMBO J.

[plbi-03-03-12-b29] Lu BY, Ma J, Eissenberg JC (1998). Developmental regulation of heterochromatin-mediated gene silencing in Drosophila. Development.

[plbi-03-03-12-b30] Csink AK, Henikoff S (1998). Large-scale chromosomal movements during interphase progression in Drosophila. J Cell Biol.

[plbi-03-03-12-b31] Wolff T, Ready DF (1993). Pattern formation in the *Drosophila* retina. In: Bate MA, editor. The development of *Drosophila melanogaster*.

[plbi-03-03-12-b32] Baker NE, Yu SY (2001). The Egf receptor defines domains of cell cycle progression and survival to regulate cell number in the developing Drosophila eye. Cell.

[plbi-03-03-12-b33] Horsfield J, Penton A, Secombe J, Hoffman FM, Richardson H (1998). decapentaplegic is required for arrest in G1 phase during Drosophila eye development. Development.

[plbi-03-03-12-b34] Lehner CF, O'Farrell PH (1989). Expression and function of Drosophila cyclin A during embryonic cell cycle progression. Cell.

[plbi-03-03-12-b35] Sage BT, Csink AK (2003). Heterochromatic self-association, a determinant of nuclear organization, does not require sequence homology in Drosophila. Genetics.

[plbi-03-03-12-b36] Mahy NL, Perry PE, Gilchrist S, Baldock RA, Bickmore WA (2002). Spatial organization of active and inactive genes and noncoding DNA within chromosome territories. J Cell Biol.

[plbi-03-03-12-b37] Solovei I, Cavallo A, Schermelleh L, Jaunin F, Scasselati C (2002). Spatial preservation of nuclear chromatin architecture during three-dimensional fluorescence in situ hybridization (3D-FISH). Exp Cell Res.

[plbi-03-03-12-b38] Tolchkov EV, Rasheva VI, Bonaccorsi S, Westphal T, Gvozdev VA (2000). The size and internal structure of a heterochromatic block determine its ability to induce position effect variegation in Drosophila melanogaster. Genetics.

[plbi-03-03-12-b39] Ahmad K, Henikoff S (2001). Modulation of a transcription factor counteracts heterochromatic gene silencing in Drosophila. Cell.

[plbi-03-03-12-b40] Csink AK, Bounoutas A, Griffith ML, Sabl JF, Sage BT (2002). Differential gene silencing by *trans*-heterochromatin in Drosophila melanogaster. Genetics.

[plbi-03-03-12-b41] Li Y, Danzer JR, Alvarez P, Belmont AS, Wallrath LL (2003). Effects of tethering HP1 to euchromatic regions of the Drosophila genome. Development.

[plbi-03-03-12-b42] Chubb JR, Boyle S, Perry P, Bickmore WA (2002). Chromatin motion is constrained by association with nuclear compartments in human cells. Curr Biol.

[plbi-03-03-12-b43] Thomson I, Gilchrist S, Bickmore WA, Chubb JR (2004). The radial positioning of chromatin is not inherited through mitosis but is established de novo in early G1. Curr Biol.

[plbi-03-03-12-b44] Walter J, Schermelleh L, Cremer M, Tashiro S, Cremer T (2003). Chromosome order in HeLa cells changes during mitosis and early G1, but is stably maintained during subsequent interphase stages. J Cell Biol.

[plbi-03-03-12-b45] Grewal SIS, Elgin SCR (2002). Heterochromatin: New possibilities for the inheritance of structure. Curr Opin Genet Dev.

[plbi-03-03-12-b46] Wallrath LL, Elgin SC (1995). Position effect variegation in Drosophila is associated with an altered chromatin structure. Genes Dev.

[plbi-03-03-12-b47] Belyaeva ES, Demakova OV, Umbetova GH, Zhimulev IF (1993). Cytogenetic and molecular aspects of position-effect variegation in Drosophila melanogaster. V Heterochromatin-associated protein HP1 appears in euchromatic chromosomal regions that are inactivated as a result of position-effect variegation. Chromosoma.

[plbi-03-03-12-b48] Fanti L, Dorer DR, Berloco M, Henikoff S, Pimpinelli S (1998). Heterochromatin protein 1 binds transgene arrays. Chromosoma.

[plbi-03-03-12-b49] Cryderman DE, Tang H, Bell C, Gilmour DS, Wallrath LL (1999). Heterochromatic silencing of Drosophila heat shock genes acts at the level of promoter potentiation. Nucleic Acids Res.

[plbi-03-03-12-b50] Gersh ES (1963). Variegation at the white locus in In(1)rst^3^. Drosoph Inf Serv.

[plbi-03-03-12-b51] Henikoff S, Dreesen TD (1989). Trans-inactivation of the Drosophila brown gene: Evidence for transcriptional repression and somatic pairing dependence. Proc Natl Acad Sci U S A.

[plbi-03-03-12-b52] Dernburg AF, Sedat JW (1998). Mapping three-dimensional chromosome architecture in situ. Methods Cell Biol.

[plbi-03-03-12-b53] Hartl DL, Nurminsky DI, Jones RW, Lozovskaya ER (1994). Genome structure and evolution in *Drosophila* Applications of the framework P1 map. Proc Natl Acad Sci U S A.

[plbi-03-03-12-b54] Hartl DL, Lozovskaya ER (1995). The *Drosophila* genome map: A practical guide.

[plbi-03-03-12-b55] Li C, Meinertzhagen IA (1995). Conditions for the primary culture of eye imaginal discs from Drosophila melanogaster. J Neurobiol.

[plbi-03-03-12-b56] Belmont AS, Braunfeld MB, Sedat JW, Agard DA (1989). Large-scale chromatin structural domains within mitotic and interphase chromosomes in vivo and in vitro. Chromosoma.

[plbi-03-03-12-b57] Paddy MR, Belmont AS, Saumweber H, Agard DA, Sedat JW (1990). Interphase nuclear envelope lamins form a discontinuous network that interacts with only a fraction of the chromatin in the nuclear periphery. Cell.

[plbi-03-03-12-b58] Bass HW, Marshall WF, Sedat JW, Agard DA, Cande WZ (1997). Telomeres cluster de novo before the initiation of synapsis: A three-dimensional spatial analysis of telomere positions before and during meiotic prophase. J Cell Biol.

[plbi-03-03-12-b59] Hiraoka Y, Swedlow JR, Paddy MR, Agard DA, Sedat JW (1991). Three-dimensional multiple-wavelength fluorescence microscopy for the structural analysis of biological phenomena. Semin Cell Biol.

[plbi-03-03-12-b60] Hiraoka Y, Sedat JW, Agard DA (1990). Determination of three-dimensional imaging properties of a light microscope system. Partial confocal behavior in epifluorescence microscopy. Biophys J.

[plbi-03-03-12-b61] Agard DA, Hiraoka Y, Shaw P, Sedat JW (1989). Fluorescence microscopy in three dimensions. Methods Cell Biol.

[plbi-03-03-12-b62] Chen H, Hughes DD, Chan TA, Sedat JW, Agard DA (1996). IVE (Image Visualization Environment): A software platform for all three-dimensional microscopy applications. J Struct Biol.

[plbi-03-03-12-b63] Zamir E, Katz BZ, Aota S, Yamada KM, Geiger B (1999). Molecular diversity of cell-matrix adhesions. J Cell Sci.

[plbi-03-03-12-b64] Sokal RR, Rohlf FJ (1981). Biometry, 2nd ed.

[plbi-03-03-12-b65] Lindsley DL, Zimm GG (1992). The genome of *Drosophila melanogaster*.

[plbi-03-03-12-b66] Lohe AR, Hilliker AJ, Roberts PA (1993). Mapping simple repeated DNA sequences in heterochromatin of Drosophila melanogaster. Genetics.

