# Supplementary material for: Cell-by-Cell Dissection of Gene Expression and Chromosomal Interactions Reveals Consequences of Nuclear Reorganization
Source: PLoS Biol. 2005 Mar 1;3(3):e67. doi: 10.1371/journal.pbio.0030067 (PMC1054879; doi:10.1371/journal.pbio.0030067)
Supplement: Supplementary file 1 [file pbio.0030067.sd001.doc]

**Protocol S1.** Anti-White Antibody Generation, and the Effects of Gene Expression, Nuclear Dimensions, and Time on Gene to Heterochromatin Distances

**1. Generation of Antibodies to White**

A unique N-terminal peptide sequence from the putative White protein sequence [1] was selected using the software program Pileup (Wisconsin Package Version 10, Genetics Computer Group (GCG), Madison, WI.) and PHDsec (http://www.embl-heidelberg.de/predictprotein/, [2,3]). This N-terminal peptide had the sequence H_3_N-MGQEDQELLIRGGSKHPSAE-COOH. This sequence was provided to Research Genetics (Huntsville, Alabama) for production of antibodies in rabbits. Antibodies were purified by immunoaffinity chromatography followed by precipitation and reconstitution at 5 mg/mL [4].

Western blot of the purified antibody against adult and larval high-speed lysates found a single band at about 70 kDa not seen when stained with PreImmune sera (Figure S1A). Immunofluorescence found that the antibody stained the posterior section of third instar eye disks (Figure S1B) in a pattern similar to a LacZ reporter gene driven by the *white* eye enhancer [5]. The anti-White antibody stained *white*-variegating lines in a mottled pattern as expected from their eye pigmentation (Figure S1C). A careful examination of cell types in the eye disk determined that White protein is first expressed only in the 8th photoreceptor (r8, Figure 5E). A careful examination of cell types in the eye disk determined that White protein is first expressed only in the 8th photoreceptor (r8, Figure S1D). Therefore the cell by cell comparison of expressing and non-expressing cells in the eye disk was between cells of the same type (r8) from different ommatidia rather than within each ommatidium. Later in development the White protein is expressed in other photoreceptors and pigment cells in the adult eye.

**2. Variegating rearrangments modify the position of variegating loci independent of gene expression**

Prior studies have demonstrated the ability of the *bw^D^* rearrangment to modify the nuclear position of *bw* in embryonic, neuroblast, and imaginal disk tissues [6,7]. This modification of nuclear position may allow a locus to interact with heterochromatin and affect later gene expression. We revisited this issue for all three variegating rearrangments. Distances between the affected locus and cis-heterochromatin were probed in wild type imaginal disk tissue and compared to the respective variegating lines (Table S1). Overall, loci on wild type chromosomes were statistically distinct from their rearranged counterparts in both dividing (anterior) and differentiated (posterior) cells. Distinctions locus-to-heterochromatin distances between wild type and variegating chromosomes are also apparent in the cumulative percentage plots presented in Figure 5. The lone exception was found with respect to the *bw* gene in wild type and *bw^D^* chromosomes; in cells anterior to the morphogenic furrow distances were not statistically significant (Table S1).

For lines with expressing cells in the imaginal disk (In(1)*rst^3^* and In(3L)BL1), wild type chromosomes were also compared to expresssing and silenced cells (Table S1). Loci on wild type chromosomes were quite different than both expressing and silenced cells from the variegating line. Data is presented graphically in the cumulative percentage plots (Figure 3). This means that even though loci in expressing cells are farther from cis-heterochromatin than in silenced cells, expressing loci do not behave as wild type.

This shows that differences in locus-to-heterochromatin distances between anterior and posterior cells are due to the chromosome rearrangement rather than changes in other criteria such as nuclear dimensions.

**3. Comparisons with random distributions of interloci distances.**

If loci from wild type chromosomes do not behave as variegating loci in expressing cells, it is possible that loci on wild type chromosomes are randomly positioned with respect to their cis-heterochromatin. Distributions of interloci distances were compared with random distributions in the following manner. Experimental data were compared to the pairwise distances measured from fifty points randomly placed per nucleus of two cell types (anterior cells vs differentiated posterior cells). Distributions of distances within each cell type were pooled for comparison to experimental populations. Monte Carlo distributions were compared to experimental data using the Mann-Whitney U test [8]. Interloci distances in expressing and silenced cells are distinct from random distributions (Table S1, results shown are from In(1)rst3).

For loci on wild-type chromosomes, the distributions were not always distinct from random distributuions (Table S1). The *white* locus on wild type chromosomes did not appear to be randomly distributed, while the *bw* locus on wild type chromosomes appeared randomly distributed relative to cis-heterochromatin. The In(3L)BL1 locus on a unrearranged chromosome was indistinguishable from a random distribution in both anterior and posterior cells of the disk.

**4. Normalization with respect to nuclear dimensions**

Previous studies have often normalized distances between an affected locus and heterochromatin for nuclear radius. Gross nuclear dimensions in the cell types presented here did not have significant effects upon observed distances. Overall, normalizing for nuclear volume or radius did not render differences between cell types insignificant (Table S2A). The lone exception were distances between anterior and posterior cells in In(1)*rst^3^* nuclei, but this was only true for a volume normalization rather than a radius. Distance between the variegating gene and heterochromatin was not correlated with nuclear volume or nuclear radius, nor did it vary between nuclei of radically different shape such as cone cells and photoreceptor cells (Table S2B). Even chromosomal loci on wild type chromosomes that do not associate with heterochromatin that might be expected to have a more volume-dependent distribution of distances showed little correlation between distance and nuclear volume or radius (not shown). The more biologically relevant determinants of distance between different loci appear to be the strength of their interaction and the dimensions of the actual chromosome.

**5. Long-range chromosomal interactions do not increase with the increasing amounts of time after exiting the cell cycle**

The examination of the gene-to-heterochromatin distance distributions of all lines show that every distribution has significant numbers of nuclei with variegating gene to centromere distances of 2 microns or more (Figure 5, D,G, and J). If interaction with heterochromatin is important for the regulation of this gene, why isn’t the chromosomal locus interacting with heterochromatin in every nucleus? One possibility is that association increases with time after exiting the cell cycle. Nuclei in the ‘tail’ of the distributions are those that most recently exited the cell cycle, while those with shorter distances exited earlier. Differentiation in the eye disk proceeds such that rows of cells closest to the furrow are youngest, while those posterior are oldest. New rows of cells are added every 1.5 hours, so each row of cells is about ninety minutes older than the previous. Therefore, differentiated cells 10 rows posterior to furrow exited the cell cycle 15 hours before cells at the furrow’s edge.

Because our unique methods preserve cell shape and position, it was straightforward to analyze each row of differentiated cells individually and compare them to one another (Figure S2A-C). Individual rows of nuclei were modeled and compared to more posterior rows in a pairwise fashion for all three lines. Data is presented both as row by row scattergrams (Figure S2D-F) and as cumulative percentage plots (Figure S2G-I). Data presented in Figure 8 has been pooled from 3 disks per line. Scattergrams are constructed as pooled data from each row, and the number of rows was the minimum number analyzed per disk. In no case were significant differences observed between different rows of cells, whether results for each row were pooled from multiple disks or looked at individually. Rows were compared to one another in pairs and were never found to be statistically unique (p > 0.05). All lines without exception showed the same association levels regardless of the row.

The fact that differentiated nuclei varying in many hours post-differentiation show the same gene-to-heterochromatin distance distributions suggests an alternative hypothesis: that almost all changes in LRCIs begin in the morphogenic furrow. This is supported by the observation that distances within the furrow are intermediate between those seen ahead of and behind the furrow, and in the case of *bw^D^*, a bimodal distribution representing both populations.

References

1 O'Hare K, Murphy C, Levis R, Rubin GM (1984) DNA sequence of the white

locus of Drosophila melanogaster. J Mol Biol 180: 437-455.

2 Rost B, Sander C (1994) Conservation and prediction of solvent accessibility in

protein families. Proteins 20: 216-226.

3 Rost B (1996) PHD: predicting one-dimensional protein structure by profilebased

neural networks. Methods Enzymol 266: 525-539.

4 Harlow E, Lane D (1988) Storing and Purifying Antibodies. Cold Spring Harbor:

Cold Spring Harbor Laboratory. 283-318 p.

5 Qian S, Varjavand B, Pirrotta V (1992) Molecular analysis of the zeste-white

interaction reveals a promoter-proximal element essential for distant enhancerpromoter

communication. Genetics 131: 79-90.

6 Csink AK, Henikoff S (1996) Genetic modification of heterochromatic

association and nuclear organization in Drosophila. Nature 381: 529-531.

7 Dernburg AF, Broman KW, Fung JC, Marshall WF, Philips J, et al. (1996)

Perturbation of nuclear architecture by long-distance chromosome interactions.

Cell 85: 745-759.

8 Sokal RR, Rohlf FJ (1981) Biometry. New York: W.H. Freeman and Company.

859 p.
