# Supplementary material for: Cell-by-Cell Dissection of Gene Expression and Chromosomal Interactions Reveals Consequences of Nuclear Reorganization
Source: PLoS Biol. 2005 Mar 1;3(3):e67. doi: 10.1371/journal.pbio.0030067 (PMC1054879; doi:10.1371/journal.pbio.0030067)
Supplement: Supplementary file 2 [file pbio.0030067.st001.doc]

Table S1: Variegating Locus-to-Heterochromatin Distances in Wild Type Cells and Random Distributions

P Values Relative toLocus^1^Disk Cell Location^2^nMedian Distance (microns)VarianceVariegating BulkVariegating expressingVariegating silencedRandom DistributionsbwAnterior942.6101.1050.1303N.R.N.R.<0.0001Posterior2191.9120.783<0.0001N.D.N.D.0.0203WhiteAnterior1012.5641.703<0.0001N.R.N.R.<0.0001Posterior3011.8861.267<0.0001<0.0001<0.0001<0.0001In(3L)BL1Anterior1172.1361.174<0.0001N.R.N.R.0.8197Posterior2042.1750.660<0.0001<0.0001<0.00010.0625RandomAnterior43582.0890.816<0.0001N.R.N.R.N.R.Posterior103382.1020.863<0.0001<0.0001<0.0001N.R.Notes: ^1^Locus refers to the same FISH probe used to visualize the affected gene as described in Materials and

Methods. Random refers to a randomly generated distance distribution as described in supplementary materials. ^2^Disk Cell Location refers to position relative to morphogenic furrow. N.D. = Not Determined, N.R.= Not Relevant.
