# Supplementary material for: Cell-by-Cell Dissection of Gene Expression and Chromosomal Interactions Reveals Consequences of Nuclear Reorganization
Source: PLoS Biol. 2005 Mar 1;3(3):e67. doi: 10.1371/journal.pbio.0030067 (PMC1054879; doi:10.1371/journal.pbio.0030067)
Supplement: Supplementary file 3 [file pbio.0030067.st002.doc]

Table S2. Effects of Nuclear Dimensions on Variegating Gene-to-Heterochromatin Distances

A. Effect of Normalization on Difference Between Cell Populations

|  | *bw^D^* | |  | *In(1)rst^3^* | |  | In(3L)BL1 | |
| --- | --- | --- | --- | --- | --- | --- | --- | --- |
|  | Ant vs Post | Cone vs Phot |  | Ant vs Post | Cone vs Phot |  | Ant vs Post | Cone vs Phot |
| Pre normalization | <0.0001 | 0.4901 |  | <0.0001 | 0.0705 |  | 0.0011 | 0.0566 |
| Volume | <0.0001 | 0.0031 |  | 0.018 | 0.1495 |  | 0.0088 | 0.5462 |
| Radius | <0.0001 | 0.1209 |  | <0.0001 | 0.4676 |  | 0.186 | 0.2987 |

B. Correlation Coefficients of Distances Relative to Nuclear Volume

|  | *bw^D^* | | |  | *In(1)rst^3^* | | |
| --- | --- | --- | --- | --- | --- | --- | --- |
|  | Anterior | Cone cell | Photoreceptor cell |  | Anterior | Cone cell | Photoreceptor cell |
| r squared | 0.031 | 0.001 | 0.033 |  | 0.011 | 0.006 | 0.0002 |
| p value | 0.0754 | 0.7283 | 0.0173 |  | 0.402 | 0.434 | 0.8043 |
|  |  |  |  |  |  |  |  |
|  | In(3L)BL1 | | |  |  |  |  |
|  | Anterior | Cone Cell | Photoreceptor cell |  |  |  |  |
| r squared | 0.006 | 0.0003 | 0.014 |  |  |  |  |
| p value | 0.5553 | 0.8722 | 0.0587 |  |  |  |  |
